# Gender and Osteopathic Representation in U.S. General Surgery Residency, 2024-2025: Divergent Patterns Across Postgraduate Training

**DOI:** 10.7759/cureus.106152

**Published:** 2026-03-30

**Authors:** Donna Mehdiyar, Samuel M Baule, Justin Nguyen, Mia A Panlilio, Hayden Flume, Allen Hanna, Aila Cordero, Julia K Shepherd, Julieanne P Sees

**Affiliations:** 1 Chicago College of Osteopathic Medicine, Midwestern University, Downers Grove, USA; 2 College of Osteopathic Medicine, Marian University, Indianapolis, USA; 3 Medicine, Edward Via College of Osteopathic Medicine, Monroe, USA; 4 College of Osteopathic Medicine, Rocky Vista University, Englewood, USA; 5 Texas College of Osteopathic Medicine, University of North Texas Health Science Center, Fort Worth, USA; 6 Medicine, Arkansas College of Osteopathic Medicine, Fort Smith, USA; 7 Dr. Kiran C. Patel College of Osteopathic Medicine, Nova Southeastern University, Fort Lauderdale, USA; 8 College of Osteopathic Medicine, University of New England, Portland, USA; 9 Haub School of Business, St. Joseph's University/American Osteopathic Association, Chicago, USA

**Keywords:** gender representation in surgery, general surgery residency, osteopathic medical graduates, osteopathic residents, postgraduate surgical training, resident demographics, surgical workforce diversity

## Abstract

Introduction

Women and osteopathic (DO) physicians remain underrepresented in the field of general surgery, from residency training to the practicing workforce. Efforts to improve diversity in general surgery residency have focused on gaps in gender and osteopathic representation; however, it remains unclear whether these patterns change consistently across postgraduate years (PGY). This study tested the hypothesis that gender and osteopathic representation follow distinct patterns across training by evaluating resident-level differences in gender representation across PGY levels and degree type, and by examining program-level associations among female representation, osteopathic representation, and historical American Osteopathic Association (AOA) affiliation in U.S. general surgery residency programs.

Methods

This retrospective cross-sectional study analyzed publicly available resident rosters from Accreditation Council for Graduate Medical Education-accredited U.S. general surgery residency programs during the 2024-25 academic year. Program websites, identified through the Fellowship and Residency Electronic Interactive Database Access, were used as the source of detailed resident rosters. Resident-level variables included postgraduate year (PGY1-5), sex, and medical degree type (MD vs. DO). Programs lacking sufficient publicly available resident-level data were excluded. Female and DO representation were compared across PGY levels using chi-square analyses and logistic regression. Program-level analyses classified programs according to whether they met or exceeded national benchmarks for female representation (49.9%) and DO representation (15.1%), based on 2024-25 national percentages of active general surgery residents. Associations between program-level female and DO representation were assessed using chi-square testing and logistic regression.

Results

Among 364 accredited programs, 292 met the inclusion criteria, representing 7,843 residents after exclusion of 319 residents with incomplete profiles. Female representation declined across training levels, from PGY1 (53.1%) to PGY5 (47.1%), with significant differences across cohorts. Increasing PGY level was associated with lower odds of being female (odds ratio (OR) = 0.93 per PGY increase, 95% CI: 0.90-0.96, p < 0.001). In contrast, DO representation remained relatively stable across PGY levels. Stratified analyses demonstrated similar female proportions among MD and DO residents, without significant interaction between degree type and PGY level. At the program level, programs with above-national DO representation were not more likely to also demonstrate above-national female representation (OR = 0.66, 95% CI: 0.41-1.08, p = 0.098). Likewise, historically AOA-affiliated programs were not more likely than non-AOA programs to meet or exceed the national benchmark for female representation.

Conclusions

In a large, national sample of general surgery residents, female representation decreased across PGY levels while DO representation remained stable. Osteopathic and gender representation were not significantly associated at the resident or program level, suggesting these dimensions of representation may be shaped by distinct mechanisms within surgical training. Efforts to improve equity in general surgery may therefore require targeted strategies addressing both osteopathic inclusion and the training-environment factors that may influence gender representation across residency.

## Introduction

Resident selection in general surgery training programs plays a crucial role in shaping the future surgical workforce, and achieving equitable diversity within the field has been an ongoing challenge. As national demand for surgeons continues to rise, with projections estimating a shortage of 11,100 to 19,900 surgeons by 2036, understanding demographic patterns within residency training has become increasingly important [1,2]. Historically, Doctor of Osteopathic Medicine (DO) applicants have matched into general surgery residency at lower rates than their Doctor of Medicine (MD) counterparts, contributing to lower representation of DO physicians across surgical subspecialties [3].

Despite these historical disparities, osteopathic representation in general surgery training has gradually increased. In 2023, DO physicians comprised 6.3% of the practicing general surgery workforce, yet filled 16.7% of categorical general surgery residency positions through the National Resident Matching Program that same year [1,4]. Longitudinal analyses have demonstrated gradual improvement in osteopathic integration, with U.S. DO applicants experiencing an approximate 0.4% annual increase in general surgery match rates between 1994 and 2023 [4,5]. However, less is known about how osteopathic representation varies across successive postgraduate training levels, where differences may reflect cohort effects, recruitment trends, or differential progression during training [2-4].

Gender representation in surgery has also received considerable attention. Although women comprised 24.9% of practicing general surgeons in 2023, they represented nearly half of active general surgery residents (49.9%) during the 2024-2025 academic year [1,2,4]. However, this representation is not uniform across training levels. National data suggest that the proportion of female residents declines from junior to senior postgraduate years (PGYs), raising questions about whether observed differences across cohorts reflect shifts in recruitment or factors affecting progression during training [2,3].

While the trajectories of osteopathic and gender representation have been studied independently, less is known about how these two dimensions of representation interact within general surgery training. In particular, it remains unclear whether DO residents are more likely than MD residents to be female, or whether residency programs with higher osteopathic representation also exhibit greater gender diversity. Program characteristics, including historical osteopathic affiliation, may influence recruitment patterns; however, the relationship between osteopathic representation and gender composition at the resident or program level remains poorly characterized [3].

Using publicly available data from the 2024-2025 academic year, this cross-sectional study examines the distribution of gender and osteopathic representation across five postgraduate training levels in U.S. general surgery residency programs. The present study hypothesizes that female representation varies across PGY cohorts, whereas osteopathic representation remains comparatively stable, and that programs with greater osteopathic representation do not necessarily demonstrate greater female representation. The study separately evaluates resident-level questions, including whether gender representation differs across PGY cohorts and whether female representation varies by medical degree type, and program-level questions, including whether programs with greater osteopathic representation also demonstrate greater female representation and whether historically American Osteopathic Association (AOA)-affiliated programs differ from non-AOA programs in their likelihood of demonstrating female representation equal to or greater than the national percentage. Together, these analyses provide an updated snapshot of demographic patterns within contemporary general surgery training and clarify whether gender and osteopathic representation within residency programs are shaped by shared or independent factors.

This article was previously presented as an oral (Quickshot) presentation at the 2025 American College of Osteopathic Surgeons Medical Student Section Fall Conference on September 19, 2025.

## Materials and methods

For this retrospective, cross-sectional analysis, U.S. Accreditation Council for Graduate Medical Education (ACGME)-accredited general surgery residency programs were identified, and categorical residents across five postgraduate classes in the 2024-2025 academic year were analyzed. The Fellowship and Residency Electronic Interactive Database Access of the American Medical Association was used to identify program websites, which served as the source of detailed resident rosters.

Resident-level variables included PGY level (PGY1-PGY5), sex (male vs. female), and medical degree type (MD vs. DO). Sex was abstracted from publicly available program website information, including names, pronouns, and/or photographs when available; when classification was uncertain, entries were reviewed by a second reviewer and only retained when consensus was reached based on verifiable information. Data were collected between October 2024 and January 2025 by eight reviewers. Programs were distributed across reviewers, and each program’s extracted dataset underwent independent verification by a second reviewer. Data were compiled in a centralized Google Sheet, and disagreements were resolved by group review, keeping only values that were directly verifiable and defaulting to more conservative classifications when uncertainty remained.

Programs were included if resident rosters were publicly available and contained sufficient information to ascertain PGY level and, at a minimum, degree type and sex for most residents. Of 364 ACGME-accredited programs identified, 72 were excluded because essential resident-level data (particularly degree type and/or sex) were not publicly available or were insufficient for reliable abstraction; this limitation most commonly affected military-affiliated programs and programs with incomplete or outdated online rosters. Individual residents were excluded if their degree type, PGY level, or sex could not be confirmed, or if they were non-categorical residents (ie, preliminary or research-year).

For program-level analyses, each program’s proportion of female residents and proportion of DO residents $(\frac{DO}{(MD+DO)}$) was calculated. Program status was categorized by comparing each calculated proportion to official percentages published in the ACGME’s Data Resource Book for the 2024-2025 academic year. Programs classified as “female-favoring” had a proportion of female residents equal to or greater than 49.9%, the national percentage of active female surgery residents from Table C.21 (page 77) of the Data Resource Book. Programs classified as “DO-favoring” had a proportion of DO residents equal to or greater than 15.1%, the national percentage of active DO surgery residents from Table C.15 (page 68) of the Data Resource Book [6].

Data organization and descriptive statistics were performed in Google Sheets. Inferential tests were conducted in Microsoft Excel (version 16.101.3; Microsoft Corporation, Redmond, WA). Associations between categorical variables were evaluated using chi-square tests (eg, sex distribution across PGY cohorts; DO vs. MD distribution across cohorts; and degree type by sex across PGY strata). Fisher’s exact test was used when expected cell counts were small. Outputs from chi-square and Fisher’s exact tests were cross-checked against reputable online calculators (eg, GraphPad QuickCalcs and an independent chi-square calculator) to confirm agreement.

To evaluate the association between PGY level and female representation at the resident level, a logistic regression model was fit with female sex as the dependent variable and PGY level as the independent variable. To evaluate whether osteopathic representation at the program level was associated with female representation, a separate logistic regression model was fit with female-favoring program status as the dependent variable and DO-favoring program status as the independent variable. Effect estimates were reported as odds ratios (ORs) with 95% confidence intervals (CIs) and two-sided p-values. Statistical significance was defined as p < 0.05.

## Results

Of the 364 ACGME-accredited general surgery residency programs listed for the 2024-2025 academic year, 292 were eligible based on the public availability of resident information on program websites. Across the full list of programs, 8,162 residents were initially identified. After excluding 316 residents with missing degree type and/or PGY level, and another three residents whose sex could not be confirmed through the program website information, the final analysis consisted of 292 programs and 7,843 residents.

Overall cohort characteristics by PGY level are displayed in Table 1. The total number of general surgery residents decreased by 34.33% from the PGY1 cohort (1,847) to the PGY5 cohort (1,375). From PGY2 (1,640) to PGY5, there was a 19.27% decrease in total residents. As PGY level increased, female representation progressively declined, while DO representation remained relatively stable across training levels (χ² = 6.06, df = 4, p = 0.19). Table 1 shows that there were 51.62% fewer female residents in the PGY5 cohort (647) than in the PGY1 cohort (981). Female residents outnumbered male residents in PGY1-3 but not in PGY4 or PGY5. A chi-square test revealed a statistically significant difference in gender proportions across the five cohorts (χ² = 24.64, df = 4, p < 0.001).

**Table 1:** Cohort Characteristics by PGY Level in U.S. General Surgery Residency Programs PGY: postgraduate year; DO: doctor of osteopathic medicine

PGY	Graduation Year	Total Residents (N)	Female, n (%)	DO, n (%)
PGY1	2029	1847	981 (53.1%)	285 (15.4%)
PGY2	2028	1640	906 (55.2%)	225 (13.7%)
PGY3	2027	1536	777 (50.6%)	206 (13.4%)
PGY4	2026	1445	716 (49.6%)	185 (12.8%)
PGY5	2025	1375	647 (47.1%)	181 (13.2%)
Total	–	7843	4027 (51.3%)	1082 (13.8%)

At the PGY level, Spearman correlation showed no statistically significant correlation between the proportion of DO residents and female residents (r = 0.57, p = 0.32), although interpretation is limited by the small number of PGY observations. At the resident level, logistic regression demonstrated a statistically significant decrease in the odds of being female with increasing PGY level (OR = 0.93 per PGY increase, 95% CI: 0.90-0.96, p < 0.001), corresponding to an approximate 7% decrease in odds with each additional PGY.

Female representation per PGY level was stratified by medical degree type, as shown in Table 2. While the proportion of female residents decreased with increasing PGY level among both DO and MD residents, there was no significant interaction between medical degree type and gender distribution across PGY levels (χ² = 0.07, df = 1, p = 0.79).

**Table 2:** Distribution of Female General Surgery Residents Across PGY Levels, Stratified by Medical Degree Type PGY: postgraduate year; DO: doctor of osteopathic medicine; MD: doctor of medicine

PGY	DO Residents (N)	Female DO, n (%)	MD Residents, (N)	Female MD, n (%)
PGY1	285	154 (54.0%)	1562	827 (52.9%)
PGY2	225	123 (54.7%)	1415	783 (55.3%)
PGY3	206	100 (48.5%)	1330	677 (50.9%)
PGY4	185	91 (49.2%)	1260	625 (49.6%)
PGY5	181	83 (45.9%)	1194	564 (47.2%)

At the program level, 102 programs met or exceeded national DO representation, of which 52 (50.98%) also met or exceeded national female representation, compared with 116 of 190 (61.05%) programs with DO representation below the national proportion (Table 3). A chi-square test of independence demonstrated no significant association between DO-favoring and female-favoring program status (χ² = 2.76, df = 1, p = 0.097). Consistent with this finding, logistic regression analysis demonstrated that DO-favoring programs were not significantly associated with female-favoring status (OR = 0.66, 95% CI: 0.41-1.08, p = 0.098)

**Table 3:** Program-Level Comparison Between DO-Favoring and Female-Favoring Status Programs were classified as DO-favoring if the proportion of DO residents was equal to or greater than the national proportion of active DO general surgery residents (15.1%), and as female-favoring if the proportion of female residents was equal to or greater than the national proportion of active female general surgery residents (49.9%) reported in the 2024-2025 ACGME Data Resource Book [6] DO: doctor of osteopathic medicine; ACGME: Accreditation Council for Graduate Medical Education

	≥ National Female Representation (N)	< National Female Representation (N)	Total
≥ National DO Representation	52	50	102
< National DO Representation	116	74	190
Total	168	124	292

The 36 programs identified as historically AOA-affiliated were no more likely than non-AOA programs to have female representation equal to or greater than the national proportion (Table 4).

**Table 4:** Program-Level Comparison Between AOA Affiliation and Female-Favoring Status χ² = 0.22, df = 1, p-value = 0.64. Programs were classified as female-favoring if the proportion of female residents was equal to or greater than the national proportion of active female general surgery residents (49.9%) reported in the 2024-2025 ACGME Data Resource Book [6] AOA: American Osteopathic Association; ACGME: Accreditation Council for Graduate Medical Education

	≥ National Female Representation (N)	< National Female Representation (N)	Total
AOA-Affiliated	22	14	36
Non-AOA	146	110	256
Total	168	124	292

## Discussion

This study evaluated whether gender representation and osteopathic representation align across U.S. general surgery training and, if not, identify where they diverge, using publicly available resident- and program-level data from the 2024-25 academic year. Three findings stand out. First, the proportion of residents who were female declined progressively with increasing PGY level, and gender proportions differed significantly across cohorts (χ² = 24.64, df = 4, p < 0.001). Second, the proportion of DO residents remained stable across PGY levels and did not differ significantly across cohorts. Third, measures of DO-favoring status were not meaningfully aligned with measures of female-favoring status at either the resident level (female DO vs. female MD by PGY) or the program level; furthermore, AOA-affiliated programs were no more likely than non-AOA programs to meet or exceed the national proportion of female general surgery residents. The downward trend in the proportion of female residents from PGY1 to PGY5 is consistent with several possible explanations, but these data cannot determine which explanation is correct. Because the study is cross-sectional rather than longitudinal, the observed pattern could reflect differential attrition by gender, cohort effects such as higher recruitment of women into more recent classes, differences in time away from training that alter apparent seniority in a single snapshot, or some combination of these factors.

One possible explanation is that structural and cultural barriers during training may differentially affect progression or retention for some residents. A recent review of surgical trainees’ perspectives on pregnancy and parenthood highlighted recurring, program-modifiable stressors, including unclear leave policies, cross-coverage strain, limited lactation support, childcare barriers, and variable mentorship; these factors were framed as issues that can adversely affect resident well-being and may contribute to delayed parenthood, stress, and attrition when support is inadequate [7]. Although the review by Bernal et al. is not specific to general surgery, its themes are relevant to residency training environments. Our data do not permit causal inference, but these factors remain plausible hypotheses for future longitudinal study. Importantly, the observed PGY gradient should not be interpreted as evidence of differences in competence or performance. In fact, population-level outcomes research has suggested that patients treated by female surgeons have similar outcomes overall and, in some analyses, slightly lower 30-day mortality compared with patients treated by male surgeons after accounting for patient, surgeon, and hospital characteristics [8]. This evidence supports the notion that persistent gender imbalances in senior ranks are more likely driven by systemic and cultural factors rather than by ability or skill.

In contrast to gender, degree representation (DO vs. MD) remained relatively stable across PGY levels. One interpretation is that DO representation is influenced more by entry-point selection processes, including application review, interview selection, and rank decisions, than by differential progression once residents are in training, at least in aggregate. Another possibility is that the single-accreditation era has standardized DO participation in categorical general surgery, making DO representation less sensitive to shifting dynamics during residency than gender representation. Nevertheless, program-level factors likely continue to shape where DO residents train. For example, in a national analysis of U.S. residency program characteristics, “former AOA” status and “osteopathic recognition” were strongly associated with higher representation of DO residents [9]. Within general surgery residency specifically, a similar association has been observed between AOA affiliation and a greater density of DO trainees [10]. These findings reinforce the idea that osteopathic representation is meaningfully patterned at the program level, reflecting history, identity, and structural signals, even if it does not vary substantially by PGY when aggregated across all programs.

A central contribution of this work is demonstrating that DO representation and female representation did not change in parallel across PGY levels and were not clearly associated at either the resident or program level. Conceptually, this distinction is important because different axes of representation may be influenced by different mechanisms. In our data, osteopathic representation remained relatively stable across PGY levels, which is compatible with a stronger role for entry-point selection processes such as application review, interview selection, and ranking decisions. By contrast, the decline in female representation with increasing PGY level suggests that factors operating before or during training may differ from those shaping osteopathic representation, although this study cannot determine the relative contributions of recruitment, progression, time away from training, or attrition.

At the PGY level, the absence of a statistically significant correlation between percent DO and percent female (based on just five PGY observations) should be interpreted cautiously because the analysis is underpowered by design; nevertheless, the direction and magnitude do not suggest a strong coupling. At the resident level, female representation decreased with increasing PGY among both DO and MD residents, and there was no evidence of a meaningful interaction between degree type and gender distribution across PGY levels. This implies that the factors producing the PGY-linked gender gradient affect residents broadly rather than operating primarily through degree-specific pathways.

At the program level, DO-favoring status was not significantly associated with female-favoring status. This null association is informative because it suggests that “DO-friendly” signals and “gender-inclusive” environments are not interchangeable. Program identity or historical osteopathic affiliation may influence DO recruitment, but that alone may not address the structural and cultural factors that shape gender representation and retention. Notably, Nikolla et al. reported that, in their dataset, percent female residents was not a strong predictor of DO representation in certain models once other program characteristics were considered, and associations differed depending on the inclusion or exclusion of former AOA programs. This underscores that female representation can be driven by a distinct set of variables from DO representation [9].

Taken together, these findings support consideration of targeted interventions rather than “one-size-fits-all” diversity strategies. If future longitudinal work confirms that female representation declines across senior PGY levels partly due to training-environment pressures, potentially relevant interventions could include more predictable and transparent leave policies, operationalized cross-coverage plans, lactation support, childcare access, and structured mentorship programs [7]. These supports are best understood as systems-level levers that may reduce avoidable friction during high-stress life and training transitions. This is particularly important in the context of broader surgical workforce concerns. Workforce modeling has projected substantial future shortfalls in general surgeons without expansion in training capacity or improved retention, highlighting supply-side constraints as a persistent vulnerability [11]. If the field is already facing a pipeline challenge, retaining trained residents through the senior years, when they are closest to independent practice, becomes both a pragmatic workforce strategy and an equity goal.

Limitations and future research directions

This study has several limitations that must be considered when interpreting the results. First, the data were derived from publicly available sources, making classification of key variables (gender, degree type, PGY level) vulnerable to website inaccuracies, asynchronous updates, outdated rosters, and incomplete profiles. A total of 319 residents were excluded from the final analysis due to missing profile data. If these omissions are not random, the reported proportions may be biased; for example, if excluded residents disproportionately belonged to a particular sex, PGY level, or degree group, the observed distributions could be under- or overestimated. Additionally, for the few residency programs whose websites did not clearly differentiate between categorical and preliminary PGY1 residents, all PGY1 residents were included, and percent changes in PGY5 counts were reported relative to both PGY1 and PGY2. The tradeoff for including as many categorical residents as possible is the potential inflation of the true categorical PGY1 resident count.

Additionally, gender was treated as a binary variable based on names, photos, and pronouns from publicly available residency website information, which does not capture the full spectrum of gender identity and may obscure important experiences. The PGY-level correlation analysis should also be interpreted cautiously because it was based on only five PGY observations and was therefore underpowered. Finally, because the analysis is cross-sectional, PGY-level differences cannot be interpreted as within-person change; longitudinal tracking is needed to separate cohort effects from true differential attrition and time-away patterns. Although the study included a large national sample of ACGME-accredited programs, generalizability is limited by the exclusion of programs with incomplete public data. Future research should prioritize longitudinal designs and examine whether concrete program-level measures, such as standardized leave and coverage processes, lactation resources, childcare access, and structured mentorship, are associated with smaller PGY-linked declines in female representation in general surgery, independent of osteopathic recruitment signals such as AOA affiliation or osteopathic recognition [7].

## Conclusions

In a large, national sample of general surgery residents, female representation declined with increasing PGY level, while DO representation remained stable. Importantly, osteopathic representation and female representation were not significantly associated at the PGY, resident, or program level, suggesting that distinct factors drive each form of representation. Efforts to improve equity in general surgery may therefore require dual strategies: targeted initiatives to enhance retention and advancement for women throughout training, alongside structural interventions, including faculty and program-level commitments, to maintain and support osteopathic inclusion.

## References

[1] Association of American Medical Colleges (2026). Association of American Medical Colleges: U.S. Physician Workforce Data Dashboard. U.S. Physician Workforce Data Dashboard.

[2] (2026). Association of American Medical Colleges: the complexities of physician supply and demand: projections from 2021 to 2036. https://www.aamc.org/media/75236/download.

[3] Cummings M (2021). The Impact of the ACGME/AOA Single Accreditation System on osteopathic surgical specialties, residents, and DO students. J Surg Educ.

[4] National Resident Matching Program (2026). Results and Data: 2024 Main Residency Match. Results and Data: 2024 Main Residency Match.

[5] Foley BL, Dougherty CE, Are C (2024). International medical graduates matching into US general surgery residency: 30-year match trends. J Surg Res.

[6] Accreditation Council for Graduate Medical Education (2026). Data Resource Book: Academic Year 2024-2025. Data Resource Book: Academic Year 2024-2025.

[7] Bernal IC, Moon SL, Hotta M, Newman MI (2024). Residents' perspectives of pregnancy and growing a family during surgical training: a review of the literature. Cureus.

[8] Wallis CJ, Jerath A, Aminoltejari K (2023). Surgeon sex and long-term postoperative outcomes among patients undergoing common surgeries. JAMA Surg.

[9] Nikolla DA, Sachdeva V, Distefano A, Bryan A, Mudrakola V, Milliron ML, Bowers KM (2025). Residency program characteristics associated with osteopathic resident representation. Cureus.

[10] Baule SM, Nguyen J, Mehdiyar D (2025). Osteopathic acceptance in general surgery residency: a five-year review of resident outcomes. Cureus.

[11] Ellison EC, Pawlik TM, Way DP, Satiani B, Williams TE (2018). Ten-year reassessment of the shortage of general surgeons: increases in graduation numbers of general surgery residents are insufficient to meet the future demand for general surgeons. Surgery.

